# Redistribution of Kv1 and Kv7 enhances neuronal excitability during structural axon initial segment plasticity

**DOI:** 10.1038/ncomms9815

**Published:** 2015-11-19

**Authors:** Hiroshi Kuba, Rei Yamada, Go Ishiguro, Ryota Adachi

**Affiliations:** 1 Department of Cell Physiology, Nagoya University Graduate School of Medicine, Nagoya 466-8550, Japan; 2 PRESTO, JST, Saitama 332-0012, Japan

## Abstract

Structural plasticity of the axon initial segment (AIS), the trigger zone of neurons, is a powerful means for regulating neuronal activity. Here, we show that AIS plasticity is not limited to structural changes; it also occurs as changes in ion-channel expression, which substantially augments the efficacy of regulation. In the avian cochlear nucleus, depriving afferent inputs by removing cochlea elongated the AIS, and simultaneously switched the dominant Kv channels at the AIS from Kv1.1 to Kv7.2. Due to the slow activation kinetics of Kv7.2, the redistribution of the Kv channels reduced the shunting conductance at the elongated AIS during the initiation of action potentials and effectively enhanced the excitability of the deprived neurons. The results indicate that the functional plasticity of the AIS works cooperatively with the structural plasticity and compensates for the loss of afferent inputs to maintain the homeostasis of auditory circuits after hearing loss by cochlea removal.

## Abstract (web summary)

Sensory deprivation in the avian brain can lead to structural changes in the axon initial segment. Here, the authors build on their previous work by showing that such homeostatic AIS plasticity also involves changes in Kv channel expression, which contributes to enhanced neuronal excitability.

## Graphical abstract

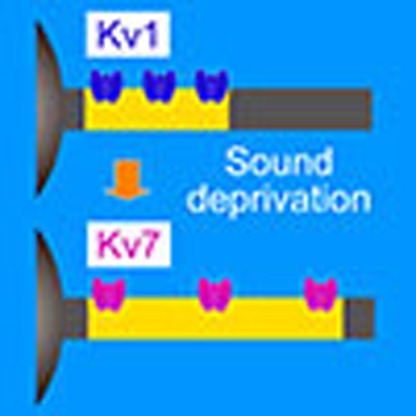

The axon initial segment (AIS) is a highly excitable axonal domain that is responsible for the initiation of action potentials (APs)^1^,^2^. This is accomplished because the domain accumulates a high density of voltage-gated Na^+^ channels (Nav) and has the lowest threshold for APs. Recently, it has been emphasized that the structural characteristics of the AIS, such as its length and its distance from the soma, are critical determinants of neuronal excitability^3^. Moreover, these characteristics are subject to change in an activity-dependent way, implying that the AIS is the site of structural plasticity and contributes to the fine regulation of neuronal activity^4^. One remarkable example is the nucleus magnocellularis (NM), an avian homologue of the mammalian anteroventral cochlear nucleus, where neurons are innervated with the auditory nerve and transmit precisely timed signals to higher auditory centres^5^. The AIS of NM neurons elongates its length after being deprived of afferent inputs^6^. This elongation increases the Na^+^ current in the axon and enhances the excitability of the neurons, thereby contributing to compensate for the loss of auditory nerve activity.

The excitability of neurons should also be influenced by the composition of ion channels at the AIS. In particular, the AIS is known to express various types of voltage-gated K^+^ (Kv) channels, such as Kv1 (Kv1.1 and Kv1.2), Kv2 (Kv2.1 and 2.2) and Kv7 (Kv7.2 and Kv7.3)^7^,^8^,^9^,^10^,^11^,^12^,^13^,^14^,^15^. Among them, Kv1 and Kv7 have low-voltage-activation characteristics and can directly affect the AP threshold^16^, raising the possibility that the elongation of the AIS increases the K^+^ current in the axon, which may counterbalance the increase in the Na^+^ current and reduce the excitability of the neurons. Accordingly, a long AIS can decrease the membrane excitability in some neurons^17^. However, the way the expression of Kv channels is regulated during the elongation of the AIS and the way this regulation affects the excitability of neurons are poorly understood.

Thus, we addressed these questions in NM neurons with immunohistochemistry, electrophysiology and computer simulation. We found that the elongation of the AIS is accompanied by a complementary change in the expression of Kv channels at the site, that is, a decrease in Kv1.1 and an increase in Kv7.2. The activation kinetics of Kv7.2 is far slower than that of Kv1.1 (ref. 16). This slow kinetics of Kv7.2 reduces shunting conductance at the AIS during AP generation, enabling the elongated AIS to trigger APs more efficiently. These results indicate that the structural and biophysical reorganizations of the AIS work synergistically and maintain neuronal excitability in the central auditory circuit after the loss of auditory inputs.

## Results

### Complementary changes in Kv channel expression

**Figure 1:**
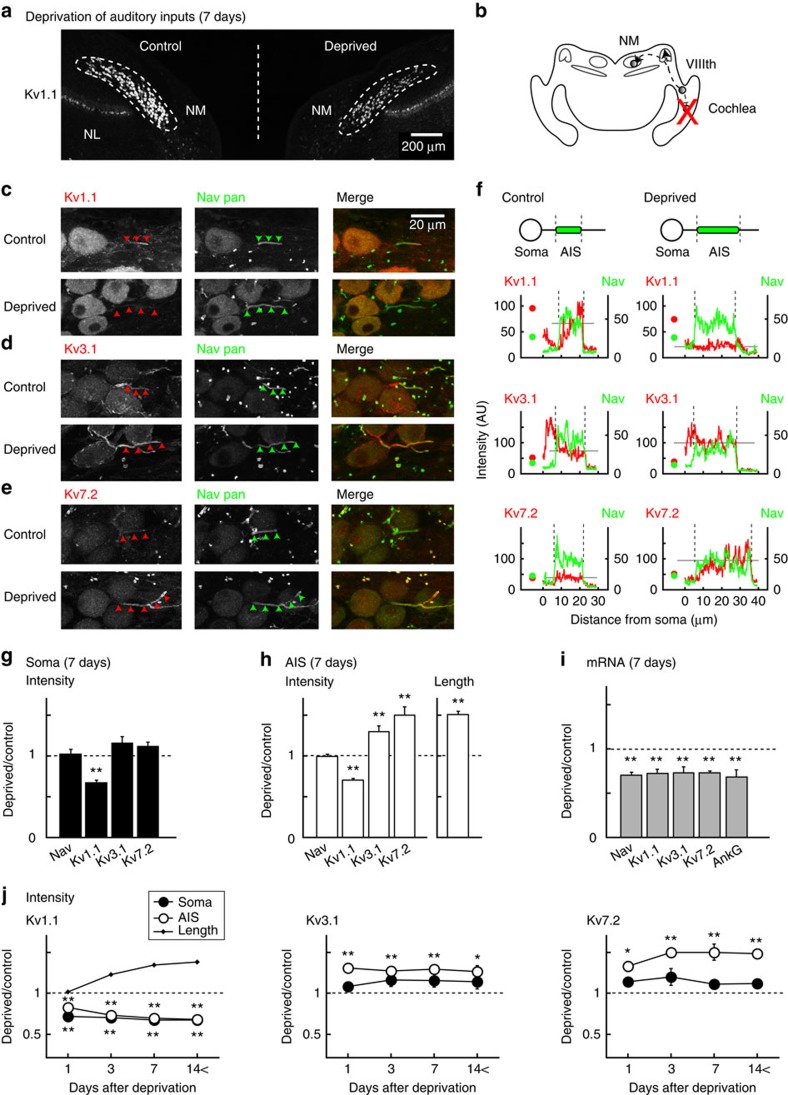
Expressions of Kv channels at the AIS in control and deprived neurons. (**a**) Macroscopic view of brainstem auditory nuclei at 7 days after auditory deprivation. Kv1.1 immunosignals were identified in NM and its target nuclei (nucleus laminaris, NL), but the signals were more prominent in control (left) than in deprived side (right) in NM. (**b**) Schematic drawing of avian auditory pathway. (**c**–**e**) NM neurons stained with Kv (left, red) and Nav pan (middle, green) antibodies were merged (right). Kv1.1 (**c**), Kv3.1 (**d**) and Kv7.2 (**e**). Control (upper) and deprived (lower) neurons were from the same slice. (**f**) Intensities of immunosignals at the soma (circles) and the axon in **c**–**e** were plotted. The AIS was identified by fibrous Nav signals (arrowheads in **c**–**e**; vertical broken lines in **f**). Average Kv intensity at the AIS is indicated by grey horizontal lines. Dot-like Nav signals correspond to nodes of Ranvier^6^ (middle). (**g**,**h**) Relative signal intensities of Nav pan, Kv1.1, Kv3.1 and Kv7.2 in the soma (**g**) and the AIS (**h**) at 7 days after auditory deprivation. Relative length of the AIS (**h**, right). Ratios were calculated in each slice and averaged for five animals (see also Supplementary Fig. 1). Kv1.1 decreased at both the soma and the AIS, whereas Kv7.2 and Kv3.1 increased only at the AIS. (**i**) Relative mRNA levels at 7 days after auditory deprivation. Three sets of experiments were conducted for individual molecules (Methods). The uniform reductions of mRNA may suggest the involvement of post-transcriptional mechanisms. (**j**) Time course of changes in signal intensity of Kv1.1 (left), Kv3.1 (middle) and Kv7.2 (right). The time course of changes in the AIS length is plotted (left, dots). Four, four, five and six chicks were used for 1, 3, 7 and 14< days after auditory deprivation, respectively. In this and subsequent figures, * and ** indicate *P*<0.05 and *P*<0.01, respectively.

Kv1.1 and Kv3.1, which mediate low- (LVA) and high-voltage-activated (HVA) K^+^ currents, respectively, are the major subtypes of Kv channels in NM neurons^18^, whereas Kv7.2 is specifically targeted to the AIS in many neurons via an interaction with ankyrin-G, a membrane scaffold protein that anchors Nav channels at the AIS^8^. Thus, we focused our analyses primarily on Kv1.1, Kv3.1 and Kv7.2 in the present study. At 7 days after the deprivation of afferent inputs, we performed immunohistochemistry and compared the distribution of Kv channels between the control and deprived sides of NM (Fig. 1a,b, Methods). We created intensity profiles of Kv (red) and Nav (green) signals along the axon and defined the AIS by a fibrous distribution of Nav signals^6^ (Fig. 1c–e arrowheads, Fig. 1f vertical broken lines, Methods). The length of the AIS was 12.1±0.3 μm in the control (*n*=117) and 18.3±0.9 μm in the deprived neurons (*n*=113; *P*<0.01, 5 animals). In both the control and deprived neurons, Kv1.1, Kv3.1 and Kv7.2 were identified. However, their distribution differed among these subtypes. In control neurons, Kv1.1 was prominent in the soma and a distal part of the AIS (Fig. 1c upper; Fig. 1f left top), whereas Kv7.2 was very weak in both the soma and the entire AIS (Fig. 1e upper; Fig. 1f left bottom). Kv3.1 was moderate at these sites, but it was more intense in the axon hillock (Fig. 1d upper; Fig. 1f left middle).

These expressions changed greatly with an elongation of the AIS after the deprivation of afferent inputs. Kv1.1 decreased in both the soma and the AIS (Fig. 1c lower; Fig. 1f right top), whereas Kv7.2 increased specifically at the AIS (Fig. 1e lower; Fig. 1f right bottom). Kv3.1 also increased at the AIS, but the extent was small, and it did not change at the axon hillock (Fig. 1d lower; Fig. 1f right middle). We also examined expressions of Kv1.2 and Kv7.3, but their immunosignals were faint at the AIS in both the control and deprived neurons.

Then, we quantified the intensity of these immunosignals at the soma and the AIS in more than 100 cells from 5 animals, and we made cumulative histograms after normalization (Supplementary Fig. 1a,b, Methods). The histograms shifted to a weaker direction at both the soma and the AIS for Kv1.1 and to a stronger direction at the AIS for Kv7.2 and Kv3.1 in deprived neurons (orange, *P*<0.01 Kolmogorov–Smirnov test). Indeed, when the relative intensity of these Kv signals was calculated, deprived neurons showed a decrease in Kv1.1 (68%) at the soma (Fig. 1g) and increases in Kv7.2 (150%) and Kv3.1 (129%) and a decrease in Kv1.1 (70%) at the AIS (Fig. 1h). Notably, the Nav signals did not differ between the control and deprived neurons at the soma (102%) or the AIS (99%; Supplementary Fig. 1a,b top; Fig. 1g,h), confirming that the elongation of the AIS does not accompany changes in the density of Nav channels at the AIS^6^. Thus, auditory deprivation not only increased the length of the AIS but also decreased Kv1.1 at the soma and the AIS while increasing Kv7.2 and Kv3.1 at the AIS in NM neurons.

### Time course of changes in Kv channel expression

The signal intensities of Kv channels showed substantial changes at 1 day after the deprivation of afferent inputs (Fig. 1j). In addition, they were almost saturated by 3 days and remained constant during our periods of observation until 28 days after deprivation. This time course was faster than that of the elongation of the AIS^6^; it was detected first ∼3 days after deprivation and took 7 days to complete (Fig. 1j left, dots). This finding may have resulted from the fact that the elongation of the AIS requires the reorganization of protein complexes, including cytoskeletons, membrane scaffolds and cell adhesion molecules^19^. Notably, the signal intensity of Kv1.1 changed almost in parallel at the soma and at the AIS (Fig. 1j left), whereas that of Kv7.2 and Kv3.1 increased specifically at the AIS (Fig. 1j middle and right). Because the messenger RNA (mRNA) levels of these channels reduced similarly in deprived neurons, the changes could be attributable to the mechanisms downstream of transcriptions, such as protein synthesis, transport or insertion into or retrieval from the plasma membrane (Fig. 1i).

### Kv currents in deprived neurons

**Figure 2:**
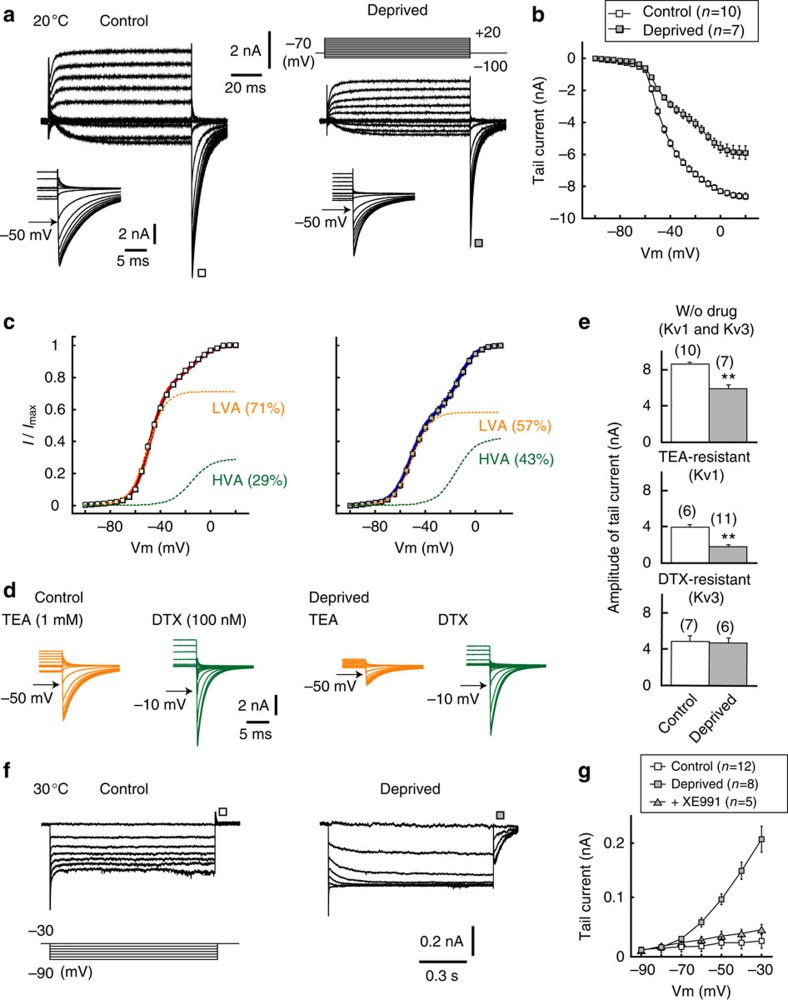
Auditory deprivation decreased Kv1 current and increased Kv7 current. (**a**) Kv1 and Kv3 currents recorded from the soma with a Cs^+^-based internal solution. Recordings were conducted at 20 °C (Methods). Voltage pulses were applied at −70 mV from −100 to +20 mV (right top). Inset, tail currents. (**b**) Current–voltage relationships of tail currents. (**c**) Voltage dependence of activation curves calculated from tail currents. The curves could be fitted by a double-Boltzmann equation in both neurons (Methods). The *V*_1/2_ and slope factor of LVA (orange) were −49.1±0.7 mV and 5.9±0.3 mV, respectively, in control neurons (*n*=10) and −51.3±1.1 mV and 6.6±0.4 mV in deprived neurons (*n*=7), whereas those of the HVA (green) were −16.5±1.6 mV and 9.7±0.5 mV in control neurons (*n*=10) and −14.9±1.2 mV and 7.9±1.3 mV in deprived neurons (*n*=7). (**d**) Tail currents in the presence of TEA (Kv3 blocker, orange) or DTX (Kv1 blocker, green). TEA- and DTX-resistant currents reflect LVA (control: 92.3±3.2%, *n*=6; deprived; 85.7±2.1%, *n*=11; *P*=0.15) and HVA (control: 93.9±1.9%, *n*=7; deprived: 94.5±1.8%, *n*=6; *P*=0.83) components, respectively. (**e**) Amplitude of tail currents at −30 mV without drugs (top), with TEA (middle) or with DTX (bottom). (**f**) Kv7 currents recorded with a K^+^-based internal solution. Recordings were conducted at 30 °C (Methods). Voltages were changed between −30 and −90 mV (left bottom). (**g**) Current–voltage relationship of tail currents. Note that the currents were blocked with XE991 (20 μM, Kv7 blocker, grey triangles). In this and subsequent figures, numbers in parenthesis are the number of cells.

We next examined the currents mediated by Kv channels under a whole-cell recording from the soma^18^ (Methods). Because NM neurons are adendritic (Fig. 1), the current should reflect that in the soma and the axon. We applied 100-ms voltage steps between −100 mV and +20 mV from a holding potential of −70 mV (Fig. 2a). The currents showed a prominent tail response at the end of voltage pulses in both the control and deprived neurons, whereas its amplitude was 27% smaller in the deprived neurons (Fig. 2a inset; Fig. 2b,e top). Notably, when we made an activation curve of the tail currents by normalizing them to the maximum value, the curve was composed of two Boltzmann components (Methods), LVA and HVA components, in both the control and deprived sides (Fig. 2c, legends for *V*_1/2_ and slope factor). Moreover, the percentage of the LVA component was smaller in the deprived side (56.6±3.3%, *n*=7) than in control side (71.0±2.1%, *n*=10). The LVA and HVA components were mediated by Kv1 and Kv3, respectively, because they were sensitive to specific blockers of Kv1 (dendrotoxin (DTX), 100 nM, green) and Kv3 (tetraethylammonium (TEA), 1 mM, orange; Fig. 2d, legends). The results indicate that Kv1 and Kv3 were predominant in both the control and deprived neurons, but Kv1 decreased after the deprivation of afferent inputs. In support of these results, when the amplitude of individual currents was evaluated pharmacologically, the TEA-resistant Kv1 current was reduced by ∼2.2 times in the deprived side (Fig. 2e middle), whereas the DTX-resistant Kv3 current did not change between the two sides (Fig. 2e bottom). Notably, the tail current disappeared in the presence of both DTX and TEA, indicating that the Kv2 current was small in the NM neurons^16^.

We then recorded the Kv7 current, which is known as the M current^20^, under blockades of Kv1 and Kv3 (Fig. 2f,g; Methods). With an increase in the hyperpolarizing pulse (1-s duration) from −30 to −90 mV, a deactivating current at the onset and an activating tail current after the pulse appeared in deprived neurons (Fig. 2f, right), but they were not observed in the control neurons (Fig. 2f, left). The activation kinetics of the tail current was very slow, with a time constant of 48.5±3.5 ms (*n*=8) at −30 mV. In addition, the amplitude of the tail current, measured just after the pulse, started to increase at approximately −70 mV (Fig. 2g, grey square). These results were compatible with observations of the Kv7 current at the AIS in the cortical pyramidal neurons^15^. Moreover, the tail current was blocked with a specific blocker of Kv7 (XE991, 20 μM), confirming that the current was mediated by Kv7 (Fig. 2g, grey triangle). These results indicate that deprivation of afferent inputs decreased the Kv1 current while increasing the Kv7 current in NM neurons. These results are consistent with the immunohistochemical findings (Fig. 1). Notably, the Kv7 current did not appear with a Kv7 activator (retigabine, 20 μM) in control neurons; the amplitude was 19.2±13.7 pA (*n*=12) and 33.6±22.8 pA (*n*=3) at −30 mV for without and with retigabine, respectively (*P*=0.64), suggesting that the deprivation of afferent inputs altered the surface expression rather than the voltage dependence of the activation of the channels.

### Reduction of Kv1 current enhances excitability

**Figure 3:**
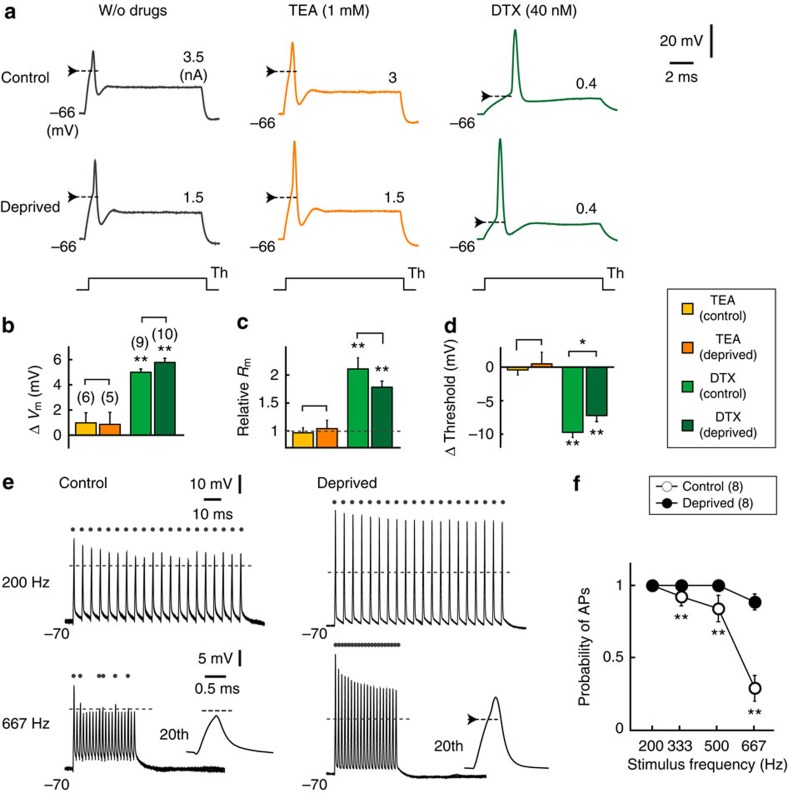
Global block of Kv1 reduced AP threshold in control and deprived neurons. (**a**) APs in response to somatic current injection. Without drugs (left), with TEA (middle) or with DTX (right). The drugs were applied to the bath. Resting membrane potential was held by injecting a current through a recording pipette. Arrows indicate AP threshold, and threshold current is indicated on the right. (**b**–**d**) Effects of the drugs on resting potential (**b**), input resistance (**c**) and AP threshold (**d**). Note that DTX affected the membrane properties similarly in both control and deprived neurons. (**e**) APs in response to a train of 20 stimuli (0.4 ms) at 200 Hz (upper) and 667 Hz (lower), which correspond to the maximum firing frequency of afferent fibres *in vivo*^53^. AP threshold and suprathreshold responses are indicated by broken lines and dots, respectively. Inset: AP at 20th stimulus. The current amplitude was 4 nA in control neurons and 3 nA in deprived neurons. (**f**) Probability of APs at each stimulus frequency. It is important to note that deprived neurons may not experience this frequency of inputs *in vivo*, because they cannot receive sound-driven inputs. Note also that the amplitude and maximum d*V*/d*t* of APs reduced with stimuli, suggesting that Na^+^ current inactivation progressed during the train. However, the extent was similar between the two sides, and its contribution to the difference in the AP probability would be small; the ratio of the maximum d*V*/d*t* between the 1st and 3rd stimuli at 667 Hz was 0.61±0.07 (*n*=4) and 0.64±0.04 (*n*=8) for control and deprived neurons, respectively (*P*=0.66). * and ** indicate *P*<0.05 and *P*<0.01, respectively. In **b**–**d**, ** on bars are comparisons against w/o drugs.

How do these complementary changes in K^+^ currents affect the excitability of NM neurons? We first compared APs during the somatic current injection between the control and deprived sides (Fig. 3a left). The deprivation of afferent inputs enhanced the excitability of NM neurons; the threshold of APs was lowered by 3 mV, the minimum current for AP generation (threshold current) was reduced by 1.8 times, and the amplitude increased by 1.7 times in the deprived neurons (Table 1). A similar reduction of the AP threshold was observed in response to synaptic inputs in the deprived neurons (6 mv, Supplementary Fig. 2). Consequently, the deprived neurons generated APs more efficiently than the control neurons did, when a train of currents was applied at a rate above 200 Hz (Fig. 3e,f). This occurred because the Na^+^ current increased with the elongation of the AIS^6^. Indeed, the maximum d*V*/d*t* of APs increased by 1.4 times in the deprived neurons. In addition, the input resistance of the deprived neurons increased (1.6 times, Table 1), which further contributed to the enhancement of excitability. The increased input resistance was due to the decreased Kv1 current (Fig. 2), as bath application of DTX (40 nM) depolarized resting potential (∼5 mV), increased input resistance (1.8–2 times) and reduced AP threshold (8–10 mV; Fig. 3a right; Fig. 3b–d green). This finding is consistent with the fact that the Kv1 current has low activation voltage, and it is activated around the resting potential (Fig. 2c, LVA). It should be noted, however, that the effects of DTX were similar between the control and deprived neurons, because the Kv1 current is large in size at the soma, and the bath application of the drug would affect the current in both the soma and the axon. In contrast, bath application of TEA (1 mM) increased the AP width slightly (1.2 times) but did not affect the resting potential, input resistance or AP threshold in either type of neuron (Fig. 3a middle; Fig. 3b–d orange), confirming that the Kv3 current has high activation voltage and activated primarily by APs (Fig. 2c, HVA). This finding further indicates that the Kv3 current accelerates the falling phase of APs in both types of neurons.

### Kv7 current stabilizes excitability of deprived neurons at the AIS

**Figure 4:**
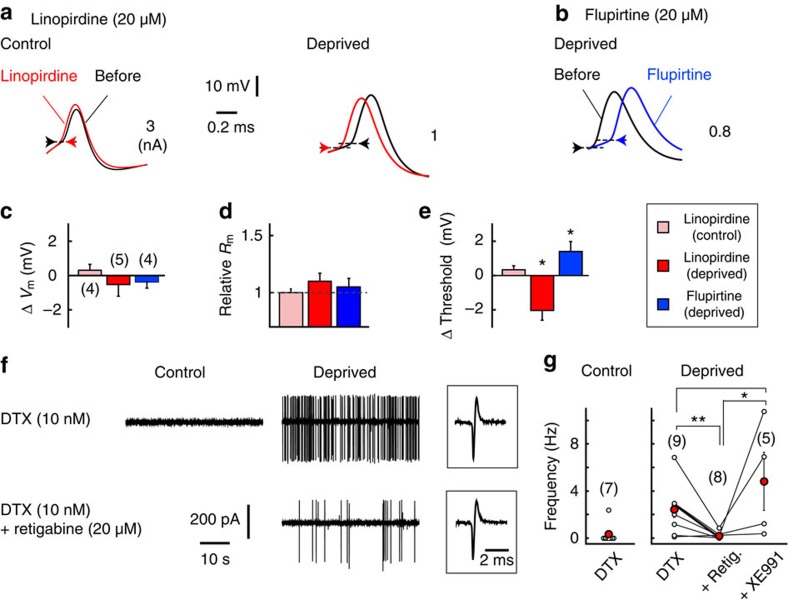
Global block of Kv7 reduced AP threshold only in deprived neurons. (**a**) APs before (black) and after (red) the bath application of linopirdine (Kv7 blocker). (**b**) APs before (black) and after (blue) the bath application of flupirtine (Kv7 activator). Arrows indicate the AP threshold. (**c**–**e**) Effects of the drugs on resting potential (**c**), input resistance (**d**) and AP threshold (**e**). Note that the drugs did not affect resting membrane properties, indicating that there were few, if any, Kv7 channels at the soma in either type of neuron. (**f**) Cell-attached recordings from control and deprived neurons. The deprived neurons showed spontaneous APs under a low concentration of DTX (right upper), and they were attenuated with retigabine (Kv7 activator, right lower). Retigabine did not affect the shapes of APs (boxes). (**g**) Frequency of spontaneous APs. The data from individual cells are plotted, and red circles indicate averages. * and ** indicate *P*<0.05 and *P*<0.01, respectively.

We next examined the effects of the Kv7 current on the excitability of NM neurons by applying a specific blocker, linopirdine (20 μM), to the bath (Fig. 4a). Linopirdine did not affect the resting potential or the input resistance of the soma in the control or deprived neurons (Fig. 4c,d), in agreement with the observations that Kv7.2 was not expressed in the soma of these neurons (Fig. 1). In contrast, linopirdine lowered AP threshold by 2 mV, and this effect was limited to deprived neurons (Fig. 4a,e). In addition, flupirtine (20 μM, Kv7 activator) had the opposite effect and increased the AP threshold by 2 mV in deprived neurons (Fig. 4b,e). These results indicate that Kv7 is activated locally at the AIS around the resting potential and stabilizes membrane excitability in deprived neurons. Consistently with this indication, under a partial block of Kv1 with a low concentration of DTX (10 nM), spontaneous APs were prominent in deprived neurons, and they were diminished with a Kv7 activator (retigabine). The effect was reversed with a Kv7 blocker (XE991; Fig. 4f,g).

### Redistribution of Kv1 and Kv7 occurs at the AIS

**Figure 5:**
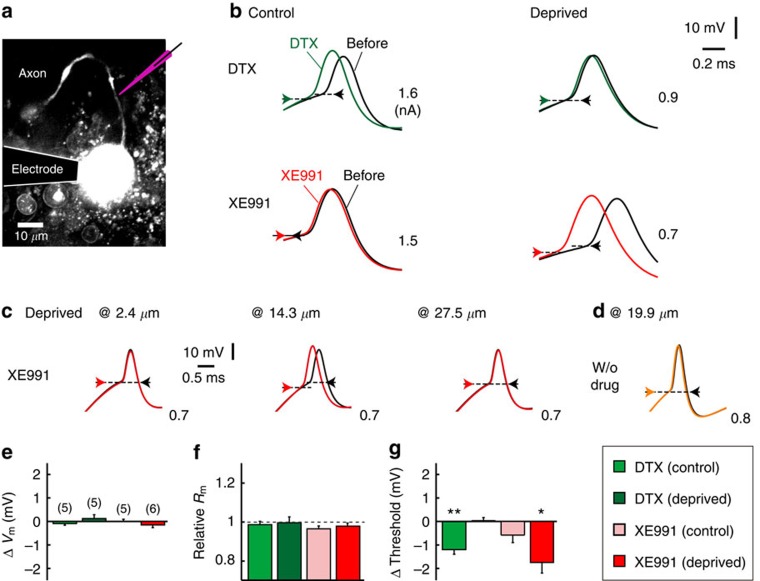
Focal block of Kv1 and Kv7 at the AIS differentially affected AP threshold in control and deprived neurons. (**a**) NM neurons filled with Alexa 594. DTX or XE991 was applied iontophoretically to the AIS with a glass pipette (purple; Methods). (**b**) APs before (black) and after an application of DTX (green, upper) or XE991 (red, lower). Arrows indicate the AP threshold. (**c**) Iontophoresis affected the AP threshold when the electrode was placed near the AIS (middle) but not when displaced proximally (left) or distally (right). (**d**) Iontophoresis did not affect the AP threshold when the electrode contained no drugs. (**e**–**g**) Effects of the drugs on resting potential (**e**), input resistance (**f**) and AP threshold (**g**). * and ** indicate *P*<0.05 and *P*<0.01, respectively.

We have shown that the Kv1 and Kv7 channels play a role in regulating the excitability of deprived neurons. However, previous experiments blocked these channels globally within the neurons and could not determine whether Kv channels at the AIS are involved in this regulation. Thus, we applied DTX or XE991 iontophoretically to the AIS with a glass electrode under an observation with a two-photon microscope (Fig. 5a, Methods). As expected from the results of bath application (Fig. 4), a focal application of XE991 reduced the AP threshold specifically in deprived neurons (1.8 mV; Fig. 5b lower; Fig. 5g). In contrast, a focal application of DTX reduced the AP threshold in the control (1.2 mV), but not in the deprived neurons (<0.1 mV; Fig. 5b upper; Fig. 5g). These results indicate that AP generation at the AIS was regulated predominantly by the Kv1 current in the control neurons and by the Kv7 current in the deprived neurons, implying that NM neurons change Kv channels at the AIS from Kv1 to Kv7 in regulating their excitability after the deprivation of afferent inputs. Importantly, these effects disappeared when the electrode was moved along the axon either proximally (2.4 μm) or distally (27.5 μm) away from the AIS (Fig. 5c) or did not include the drugs (Fig. 5d). In addition, a focal application of DTX did not affect the resting potential or input resistance in either type of neuron (Fig. 5e,f), confirming that the drugs blocked the Kv channels at the AIS. These findings further indicate that the effects of bath-applied DTX on the parameters were due primarily to a blockage of Kv1 in the soma (Fig. 3a–c).

### Redistribution of Kv1 and Kv7 in model neurons

**Figure 6:**
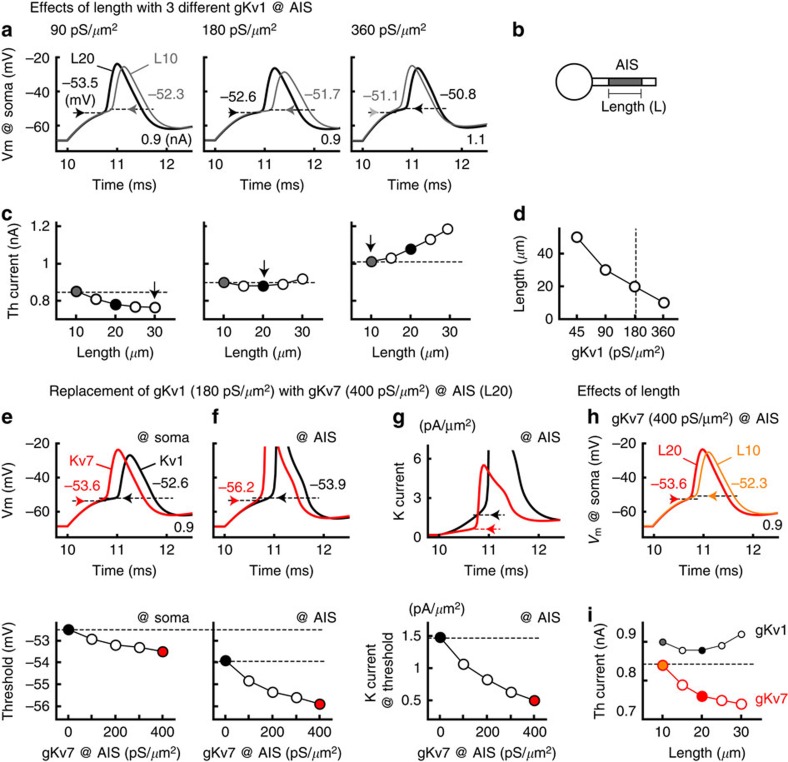
Replacement of Kv1 with Kv7 at the AIS increased excitability in a model neuron. (**a**) Effects of AIS length on APs in three different Kv1 conductances (gKv1) at the AIS. The APs from AIS length (L) of 10 μm (L10, grey) and 20 μm (L20, black) were overlaid; 90 pS μm^−2^ (left), 180 pS μm^−2^ (middle) and 360 pS μm^−2^ (right). Horizontal arrows indicate the AP threshold. A somatic current injection (20 ms) was made to induce APs; the amplitude is specified on the lower right. (**b**) Schematic drawing of model neuron (Methods). (**c**) Relationship between threshold current and AIS length. Note that the AIS length affected the threshold current differentially depending on gKv1 at the AIS. Vertical arrows indicate the minimum threshold current. (**d**) AIS length that provides a minimum threshold current negatively correlated with gKv1. (**e**–**g**) Effects of replacing gKv1 (180 pS μm^−2^, black) with gKv7 (400 pS μm^−2^, red) at the AIS. The gKv1 and gKv7 were changed to make total K^+^ current constant at the rest (Methods). The top panels show the APs at the soma (**e**) and at the AIS (**f**) and the K^+^ current at the AIS (**g**). The threshold (**e**,**f**) and K^+^ current at threshold (**g**) are indicated by horizontal arrows and are plotted against ratio of gKv7 (bottom). Note that the replacement lowered the threshold at both the soma and the AIS and reduced the K^+^ current at threshold at the AIS. (**h**) Effects of AIS length on APs in a model with gKv7 (400 pS μm^−2^) at the AIS. The APs from the AIS length of 10 μm (L10, orange) and 20 μm (L20, red) are overlaid. (**i**) The threshold current decreased monotonically with the AIS length in the model with gKv7 (400 pS μm^−2^, red), and it was systematically smaller than that in the model with gKv1 (180 pS μm^−2^, black, from **c** middle). The replacement of gKv1 with an equal density of gKv7 (180 pS μm^−2^) further augmented the reduction of the threshold current. It is important to note that replacement or reduction of gKv1 at the AIS did not affect input resistance of the soma.

We next asked how the Kv1 current at the AIS affects the excitability of NM neurons during the elongation of the AIS using a multiple-compartment model (Fig. 6b, Methods). The AIS of the model was initially set to have a Kv1 conductance (gKv1) of 180 pS μm^−2^ and no Kv7 conductance (gKv7; Fig. 6a middle). The model showed that the AP threshold was critically dependent on the length of the AIS and decreased by 0.9 mV when the AIS was elongated (Methods) from 10 μm (L10, grey) to 20 μm (L20, black) in the control and deprived neurons, respectively (Fig. 1). The effect of the elongation was pronounced when gKv1 at the AIS was halved (1.2 mV, 90 pS μm^−2^; Fig. 6a left); the effect was reversed and AP threshold increased when gKv1 at the AIS was doubled (0.3 mV, 360 pS μm^−2^; Fig. 6a right). These results may reflect the fact that an elongation of the AIS increases the total Na^+^ current at the AIS, which enables neurons to overcome the effects of conductance and capacitance loads of the soma on the AIS, but simultaneously increases the total K^+^ current at the AIS, thus preventing AP generation. The excitability of neurons is determined as a balance of these two factors^17^. Consistent with this idea, when the threshold current was plotted against length of the AIS, it showed a nonmonotonic pattern with gKv1 of 180 pS μm^−2^ at the AIS (Fig. 6c middle). It monotonically decreased with 90 pS μm^−2^ (Fig. 6c left) and increased with 360 pS μm^−2^ (Fig. 6c right). Accordingly, the AIS length that provides the lowest threshold current (Fig. 6c arrows) showed an inverse correlation with gKv1 at the AIS (Fig. 6d). A reduction of gKv1 at the soma lowered the threshold current but maintained this inverse correlation. Thus, an elongation of the AIS differentially affects membrane excitability in a manner dependent on gKv1 at the AIS and becomes disadvantageous for neurons with large gKv1 at the AIS. This finding supports the idea that the reduction of gKv1 at the AIS is critical in the enhancement of excitability during AIS elongation in deprived neurons.

Then, how does the complementary change in Kv1 and Kv7 at the AIS contribute to the excitability of the deprived neurons? We replaced gKv1 (black) with gKv7 (red) at the AIS (20 μm) in the model (Fig. 6e–g, Methods). The replacement substantially increased the excitability of the model; it lowered the AP threshold by 1.0 mV at the soma (from −52.6 to −53.6 mV; Fig. 6e) and by 2.3 mV at the AIS (from −53.9 to −56.2 mV; Fig. 6f). Accordingly, the amplitude and the maximum d*V*/d*t* of the APs increased by 11.4% (from 26.3 to 30.1 mV and from 178.1 to 204.2 V s^−1^, respectively; Fig. 6e top), and the threshold current decreased by 13.7% from 0.88 (filled black circle) to 0.76 nA (filled red circle; Fig. 6i) after the replacement. These results occurred because the activation kinetics of Kv7 was far slower than that of Kv1 (Fig. 2), which reduced the K^+^ current activated around the AP threshold at the AIS (Fig. 6g). Consequently, the threshold current monotonically decreased with the AIS length (Fig. 6i red), and the AP threshold decreased by 1.3 mV with AIS elongation from 10 (orange) to 20 μm (red; Fig. 6h) in the model with gKv7. Thus, the replacement of Kv1 with Kv7 at the AIS was particularly beneficial in augmenting the excitability of deprived neurons during the elongation of the AIS.

## Discussion

We have shown that the deprivation of afferent inputs not only elongated the AIS but also changed expressions of Kv channels and their currents in NM neurons; Kv1.1 decreased at the soma and the AIS, whereas Kv7.2 increased at the AIS (Figs 1 and 2), resulting in a switching of dominant Kv subtypes at the AIS from Kv1.1 to Kv7.2 in deprived neurons (Figs 3, 4, 5). Due to the slow activation kinetics of Kv7, the changes in Kv subtypes enabled the neurons to reduce the shunting current around the AP threshold (Fig. 6), which, along with the elongation of the AIS, effectively lowered the AP threshold and enhanced the excitability of the deprived neurons.

Various types of Kv channels, such as Kv1.2, Kv2.1, Kv3.1 and Kv4.2, show activity-dependent changes in their activation and/or surface expression, which contribute to homeostatic control of neuronal output^21^,^22^,^23^,^24^,^25^. Recently, it was revealed that Kv channels at the AIS are the target of activity-dependent changes in their activation; Kv1 channels at the AIS are inactivated during high levels of activity and broaden APs in cortical pyramidal neurons^9^,^10^,^26^, whereas Kv7 channels at the AIS are suppressed with cholinergic activity and induce an enhancement of excitability in hippocampal granule cells^27^.

In the present study, we further showed that Kv channels at the AIS undergo activity-dependent alterations in their expression. Moreover, these alterations occur in a manner specific to Kv subtypes; Kv1.1 decreases and Kv7.2 increases with deprivation of afferent inputs, leading to a complementary change in the two Kv channels at the AIS. Because both Kv1 and Kv7 have low activation voltages^16^, they are activated around the resting potential. However, their activation kinetics differs, allowing the channels to regulate AP generation differently; that is, Kv1 with fast kinetics activates rapidly with a slight membrane depolarization and suppresses AP generation actively, whereas Kv7.2 with slow kinetics activates far less during the depolarization and suppresses AP generation rather passively. Thus, NM neurons lower the AP threshold effectively with the reduction of Kv1.1 while stabilizing the resting potential with the compensatory increase in Kv7.2 at the AIS after the deprivation of afferent inputs. This may indicate that these complementary changes of Kv1.1 and Kv7.2 at the AIS could be a mechanism for regulating excitability with minimal effects on the resting potential. The absence of Kv7 in control neurons might be preferable because the slow Kv7 current could accumulate and reduce excitability during sound-driven high-frequency inputs in the neurons. This accumulation might be further augmented by depolarizing GABAergic inputs in the neurons^28^. Notably, Kv3.1 increased slightly at the AIS in deprived neurons (Fig. 1h), which may further compensate for the reduction of Kv1.1 in the repolarization of APs.

The complementary regulation of Kv channels was also reported at the soma of neurons in the hippocampus of mice and the medial nucleus of a trapezoid body^29^; prolonged synaptic stimulation decreases the Kv3 current and increases the Kv2 current via channel phosphorylation. Because Kv2 has a slightly lower activation voltage and slower kinetics than Kv3, this switching causes the cumulative activation of Kv2, promotes the repolarization of APs and augments AP generation during the stimulation. These findings suggest that a complementary regulation of multiple Kv channels could be a universal mechanism for neurons to adjust their activity efficiently and securely without drastically influencing their homeostasis. Because the AIS has a strong effect on neuronal excitability, this strategic regulation of Kv channels at the AIS would be advantageous in balancing the excitability.

Both structural and biophysical properties of the AIS are critical in determining excitability of neurons. The length and location of the AIS vary among different neuronal types^30^,^31^ and can be altered in an activity-dependent manner^6^,^32^,^33^,^34^,^35^. The types, density and localization of ion channels are also determined in a cell-type-specific manner at the AIS in various neurons^12^. Notably, these structural and biophysical properties of the AIS interact and should be coordinated appropriately^17^. In the present study, we showed that the expression of Kv1.1 was reduced before the elongation of the AIS, and this reduction was critical in enhancing the excitability of deprived neurons. Indeed, the enhancement of excitability diminished when the AIS became longer than 20 μm with a constant gKv1 at the AIS (Fig. 6a,c middle, 180 pS μm^−2^). This occurred because the AIS length that provides a high neuronal excitability became systematically shorter with an increase in gKv1 at the AIS in our model (Fig. 6d). This finding is reasonable because the elongation of the AIS would increase total number of Kv channels at the AIS and the amount of charges required for the generation of APs, if their density remained constant. This indicates that the elongation of the AIS may not be sufficient to explain the deprivation-induced changes in neuronal excitability depending on gKv1 at the AIS, and further emphasizes the importance of the synergistic control of the structural and biophysical properties of the AIS in the adjustment of neuronal activity.

Recently, it was revealed that localizing Nav channels in the axon at some distance from the soma enhances AP generation in some neurons, because the distance reduces effects of electrical loads of the soma on the process^17^,^30^,^36^. In addition, the optimum distance depends on patterns of synaptic inputs, and can be altered dynamically during the inputs^17^,^30^,^37^. This occurred because synaptic potentials attenuate during passive propagation along the axon and affect the extent of Nav-channel inactivation in a manner dependent on the distance. Indeed, inactivation of Nav channels progressed during high-frequency AP generation in NM neurons, but importantly, the extent did not change after auditory deprivation (Fig. 3e), suggesting that the AIS elongation may not affect greatly the short-term modulation of AP generation. This finding could be due to the fact that the elongation (<10 μm) was far smaller than the length constant of the proximal axon (>100 μm)^9^,^10^,^15^.

The deprivation of afferent inputs altered the density rather than the activation of Kv channels at the AIS in NM neurons. Notably, the change in Kv7.2 did not correlate with that of its mRNA (Fig. 1), suggesting an involvement of post-transcriptional mechanisms in the process. In particular, Kv7.2 increased specifically at the AIS, which may have been mediated by a mechanism operating locally at the AIS, such as insertion or retrieval at the membrane. Notably, Kv7 channels have the same ankyrin-G-binding motif as Nav channels^8^; ankyrin-G plays a role in the axonal targeting of Nav channels via direct binding with a motor protein, KIF5B^38^, raising the possibility that a similar mechanism underlies the targeting of Kv7 to the AIS. However, in NM neurons, the density of Nav channels did not change, and additional mechanisms are needed to explain the regulation of Kv7. The axonal targeting of Kv1 involves several molecules, including motor proteins such as KIF5B and KIF3A and the postsynaptic density protein 93 (refs 39, 40, 41), and it can be modulated by cyclin-dependent kinase^42^. In NM neurons, however, the expression of Kv1.1 decreased in both soma and the AIS, and the extent was almost the same between the two locations. In addition, the mRNA of Kv1.1 similarly decreased, suggesting that the reduction of Kv1.1 was mediated at the level of transcription or by other global mechanisms, such as protein synthesis and/or transport to the plasma membrane.

Although precise mechanisms remain unknown, alterations of these Kv channels might be triggered via changes in [Ca^2+^]_i_. Indeed, NM neurons elevated [Ca^2+^]_i_ within an hour after deprivation of afferent inputs^43^, which was followed by reductions of Kv1 at the soma^44^. Consistently, the elevation of [Ca^2+^]_i_ decreased the Kv1 current via the subsequent elevation of cyclic AMP and the activation of the CREB pathway^45^, whereas the axonal targeting of Kv7 was increased by Ca^2+^-dependent mechanisms via calmodulin^46^. Moreover, the structural plasticities of the AIS are known to depend on [Ca^2+^]_i_ (refs 32, 47). Thus, Ca^2+^ could be a key molecule to mediate the coordination of structural and biophysical changes in the AIS in NM neurons.

Afferent inputs to NM neurons are driven at high rates, and the deprivation of these inputs causes cell death in a subset of NM neurons^48^, suggesting that the appropriate adjustment of neuronal activity is critical in the maintenance and survival of auditory neurons. Importance of homeostatic control of neuronal excitability in auditory system is supported by the recent findings that partial or transient hearing loss causes compensatory increase of excitatory receptors (GluA2/3) and decrease of inhibitory receptors (GlyRα1) in mammalian cochlear nucleus^49^. Notably, the deprivation of the peripheral drive enhances excitability and restores activity via a compensatory decrease in the Ca^2+^-activated K^+^ current in vestibular nucleus, supporting the pivotal role of intrinsic plasticity in the homeostasis of the sensory system^50^. In the present study, we showed that the elongation of the AIS in combination with the decrease in Kv1.1 expression effectively enhances the membrane excitability of NM neurons after the deprivation of afferent inputs. This finding indicates that the structural and functional plasticities of the AIS are well coordinated and work cooperatively to compensate for the loss of afferent activity in NM neurons. Because the AIS is the site of AP generation, this coordination would be an efficient way to regulate neuronal activity and maintain the homeostasis of auditory circuits after hearing loss.

## Methods

### Animals

Chickens (*Gallus domesticus*) in post-hatch days 1–29 (P1–29) were used for immunohistochemistry and electrophysiology. The experimental animals' care was in accordance with the Regulations on Animal Experiments in Nagoya University, and all experiments were approved by the institutional committee.

### Operations

The deprivation of unilateral cochlea was performed as described previously^6^. Chicks of P1 were anesthetized with a subcutaneous injection of either chloral hydrate (0.2 g kg^−1^, Nacalai, Japan) or urethane (0.5 g kg^−1^, Polysciences, USA), and the unilateral basilar membrane was removed with fine forceps. Following survival periods of 1–28 days, the animals were used for experiments. Most experiments were conducted in neurons with a high tuning frequency (2.5–4 kHz).

### Immunohistochemistry

The mouse monoclonal Nav pan antibody (5 μg ml^−1^, Sigma), the rabbit polyclonal Kv1.1 antibody (1 μg ml^−1^, Alomone), the Kv1.2 antibody (1 μg ml^−1^, Alomone), the Kv3.1b antibody (1.5 μg ml^−1^, Sigma), the Kv7.2 antibody (1.1 μg ml^−1^, Sigma) and the Kv7.3 antibody (1.5 μg ml^−1^, Alomone) were used for immunohistochemistry. Detailed immunostaining procedures were described previously^6^. Briefly, chicks (P1–29) were perfused transcardially with a periodate-lysine–paraformaldehyde fixative (ml g^−1^ body weight): 2% (w/v) paraformaldehyde, 2.7% (w/v) lysine HCl (Nacalai), 0.21% (w/v) NaIO_4_ and 0.1% (w/v) Na_2_HPO_4_. The brainstem was post-fixed for 4 h at 4 °C. After cryoprotection with 30% (w/w) sucrose in PBS, coronal sections (30 μm) were obtained. The sections were incubated overnight with the primary antibodies and then with Alexa-conjugated secondary antibodies (10 μg ml^−1^, Life Technologies) for 2 h and were observed under a confocal laser-scanning microscope (FV1000, Olympus). Confocal sections were captured with the same microscope settings in both the control and deprived sides. Series of sections were Z-stacked at a step of 1 μm with a maximum intensity projection, and measurements were performed as described previously^6^. Most AIS ranged within 2–3 μm along the *z* axis in both control and deprived sides. The signal intensity at the AIS was the average along the entire AIS, whereas that at the soma was the average in cytosolic regions (>50 μm^2^) except the nucleus. The signals were compared between the control and deprived sides in each slice. The tissue turbidity in NM did not affect the comparisons substantially, because when slices were incubated with fluorescent beads (1 μm diameter, 580–605 nm, Life Technologies; 1.0 × 10^5^ particles per microlitre), the signals were similar between the control and deprived sides; the ratio was 1.03±0.03 (*P*=0.40, 4 animals).

### Quantitative real-time PCR

Seven days after the deprivation of unilateral cochlea at P1, the brainstem was removed^6^. NM was excised with a fine needle under an observation with a dissecting microscope, and tissues from six animals were pooled for each set of experiments. Total RNA was extracted from control and deprived sides using a NucleoSpin RNA XS kit (TaKaRa). The quality and concentration of the extracted RNA were checked with agarose gel electrophoresis and measured with NanoDrop 2000 (Thermo Scientific), and complementary DNA was synthesized using ReverTra Ace qPCR RT kit (Toyobo). The mRNA levels for Kv1.1, Kv3.1, Kv7.2, Nav1.6, Ankyrin-G and β-actin were quantified using THUNDERBIRD SYBER qPCR Mix (Toyobo) with a StepOne Real-Time PCR system (Applied Biosystems). The primers used are listed in Table 2. The cycle threshold data of individual molecules were analysed with β-actin as an internal standard, and they were compared between the control and deprived sides in each experiment.

### Electrophysiology

Five to ten days after the deprivation of unilateral cochlea at P1, coronal brain slices (200–250 μm) were obtained, as described previously^6^. During the experiments, slices were perfused with an artificial cerebrospinal fluid (concentrations in mM: 125 NaCl, 2.5 KCl, 26 NaHCO_3_, 1.25 NaH_2_PO_4_, 2 CaCl_2_, 1 MgCl_2_, and 17 glucose, 0.04 bicuculline, 0.02 CNQX, pH 7.4). Recordings were made using a patch-clamp amplifier (Multiclamp 700B, Axon)^6^, and pipettes had a resistance of 3–4 MΩ (3-μm tip diameter) when filled with a K^+^-based internal solution (113K-gluconate, 0.1 EGTA, 14 Tris_2_-phosphocreatine, 4Na_2_-ATP, 0.3 Tris-GTP, 9 HEPES-KOH, pH 7.2). The current clamp recording was made at 38–40 °C, the physiological temperature of birds, with the K^+^-based internal solution. The recording of Kv1 and Kv3 currents was made at 20 °C with a Cs^+^-based internal solution (in mM: 155 CsMeSO_3_, 5 NaCl, 3 MgCl_2_, 0.2 EGTA, 10 HEPES-KOH, pH 7.2), while [K^+^]_o_ was increased to 5 mM, [Ca^2+^]_o_ decreased to 0.5 mM, and CdCl_2_ (1 mM), NiCl_2_ (0.5 mM) and TTX (1 μM) were added to the bath. These greatly improved quality of the voltage clamp and enabled the reliable recording of the Kv currents in the NM neurons^18^. The reversal potential of the currents was −26.1±1.1 mV (*n*=6) in the control and −25.9±0.4 mV (*n*=4) in the deprived neurons (*P*=0.92). These values were similar to the theoretical value (−28.6 mV) calculated from a permeability ratio of 0.1 between Cs^+^ and K^+^ (ref. 18). The Kv7 current was recorded at 30 °C with the K^+^-based internal solution, while 4-aminopyridine (2 mM), CsCl (1 mM), CdCl_2_ (1 mM), NiCl_2_ (0.5 mM) and TTX (1 μM) were added to the bath. The cell-attached recording of spontaneous activity was performed at 38–40 °C using a pipette filled with the artificial cerebrospinal fluid, and bicuculline and CNQX were excluded from both the internal and external solutions. The electrode capacitance and series resistance (3–10 MΩ) were estimated and compensated electronically up to 95%. The liquid junction potential (3.1–11.0 mV) was corrected after the experiments. The data were sampled at 100 kHz and were low-pass filtered at 10 kHz. The parameters of spikes were analysed as described previously^6^. The voltage-dependent activation curve of Kv currents was fitted by a double Boltzmann equation; *I*/*I*_max_=*A*_1_ × {1+exp[−(*V*_m_−*V*_1/2_1)/S1]}+*A*_2_ × {1+exp[−(*V*_m_−*V*_1/2_2)/S2]}, where *I* is the tail current amplitude, *I*_max_ is the maximum tail current amplitude, *V*_m_ is the membrane potential, *A*_1_ and *A*_2_ are ratios, *V*_1/2_1 and *V*_1/2_2 are half-activation voltages, and S1 and S2 are slope factors of individual Boltzmann components. The drugs for electrophysiology were purchased from Tocris (bicuculline, CNQX, XE991, linopirdine and flupirtine), Toronto Research Chemicals (retigabine) and Wako (TTX).

### Iontophoresis

The neurons were filled with Alexa 594 (20 μM, Life Technologies) through a recording pipette, and the drugs were applied with another glass pipette under an observation with a two-photon microscope (FV1000MPE, Olympus). The pipette contained Alexa 594 (20 μM) and either DTX (400 nM, Alomone) or XE991 (200 μM, Tocris), and it was placed on the axon at 14.0±0.9 μm (*n*=4) and 17.8±0.7 μm (*n*=6) away from the soma for control and deprived neurons, respectively. The iontophoretic pulse (20 ms) was applied before a somatic depolarization with a 10-ms separation. The effects disappeared when the pipette did not include the drugs, or it was displaced from the AIS.

### Computational model

Neuronal modelling and simulation were performed with NEURON 5.8, as described previously^31^. The model consisted of multiple sections: a soma (20 μm in length and diameter) and an axon with an axon hillock (10 μm length), an AIS (20 μm length), 10 myelinated internodes and 10 nodes of Ranvier. The Nav and Kv currents were simulated using models available on the web-accessible Model DB^51^. Accession #37857 for Nav current^52^, accession #3434 for Kv1 and Kv3 currents^18^ and accession #114394 for Kv7 current^15^. The *V*_1/2_ and slope factor for activation were −58 mV and 10 mV for the Kv1 current, −19 mV and 9 mV for the Kv3 current and −30 mV and 9 mV for the Kv7 current, respectively. The Nav, Kv1 and Kv3 currents were incorporated into the soma, the AIS and the nodes; gNa, gKv1 and gKv3 were 100 pS μm^−2^, 60 pS μm^−2^ and 60 pS μm^−2^ in the soma and 14,000 pS μm^−2^, 180 pS μm^−2^ and 180 pS μm^−2^ in the AIS and the nodes, respectively. The Kv3 current was also included at the axon hillock (180 pS μm^−2^). Although gKv1 and gKv3 were adjusted to mimic the observations in voltage-clamp recordings and immunohistochemistry, the model showed slightly higher input resistances and lower threshold currents. The length of the AIS was altered with constant gNav and gKv at the AIS, implying that changes in the AIS length altered the total conductance at the AIS. When gKv1 was replaced with gKv7 at the AIS, gKv7 was set to make a total K^+^ current at the rest constant (0–400 pS μm^−2^). The temperature was 40 °C. The time step of the calculation was 12.5 μs.

### Statistics

The statistical significance was determined with a paired or unpaired Student's *t*-test, unless otherwise stated. The equality of the variances was tested with an *F*-test. The values are presented as the mean±s.e. (*n*=number of cells).

## Additional information

**How to cite this article:** Kuba, H. *et al.* Redistribution of Kv1 and Kv7 enhances neuronal excitability during structural axon initial segment plasticity. *Nat. Commun.* 6:8815 doi: 10.1038/ncomms9815 (2015).

## Supplementary Material

Supplementary Information Supplementary Figures 1-2

## Figures and Tables

**Table 1:** Parameters of action potentials in control and deprived neurons.

	**Control (*****n*****=14)**	**Deprived (*****n*****=21)**
Threshold current (nA)	3.39±0.21	1.86±0.15*
Threshold potential (mV)	−42.9±0.8	−46.2±0.8*
Amplitude (mV)	21.3±1.3	37.5±1.6*
Half-width (ms)	0.18±0.01	0.21±0.01*
Max. d*V*/d*t* (V s^−1^)	199.3±10.4	287.1±9.1*
Mini. d*V*/d*t* (V s^−1^)	−206.0±6.2	−258.2±7.6*
*R*_m_ (MΩ)	9.6±0.8	15.1±1.0*
*V*_m_ (mV)	−66.3±1.0	−66.5±0.6

*R*_m_: input resistance.

*V*_m_: resting membrane potential.

^*^indicates *P*<0.01.

**Table 2:** Primer sets used in this work.

Kv1.1	Fwd: 5′-AGCTGAAGGAGGACAAGGAG-3′	Rev: 5′-AGAACCAGATAATGCAAAGGG-3′
Kv3.1	Fwd: 5′-CGCATCTGGGCGCTCTTTG-3′	Rev: 5′-TAAATCTCTCGTGGGTCTCG-3′
Kv7.2	Fwd: 5′-ACAAGTACCCCCAGACCTG-3′	Rev: 5′-AGAGCAAATCCCGAACCTAAA-3′
Nav1.6	Fwd: 5′-ATTTCAGCAGCGATACAGACC-3′	Rev: 5′-CCTCCACAGGGACTTCTTC-3′
AnkG	Fwd: 5′-GGTGACGACGAAGTGTTTGA-3′	Rev: 5′-GCTGGAGTTGTATCGGGTGT-3′
β-Actin	Fwd: 5′-CAGACATCAGGGTGTGATGG-3′	Rev: 5′-CTTTTGCTCTGGGCTTCATC-3′
