# Supplementary material for: Redistribution of Kv1 and Kv7 enhances neuronal excitability during structural axon initial segment plasticity
Source: Nat Commun. 2015 Nov 19;6:8815. doi: 10.1038/ncomms9815 (PMC4673506; doi:10.1038/ncomms9815)
Supplement: Supplementary Information — Supplementary Figures 1-2 [file ncomms9815-s1.pdf]

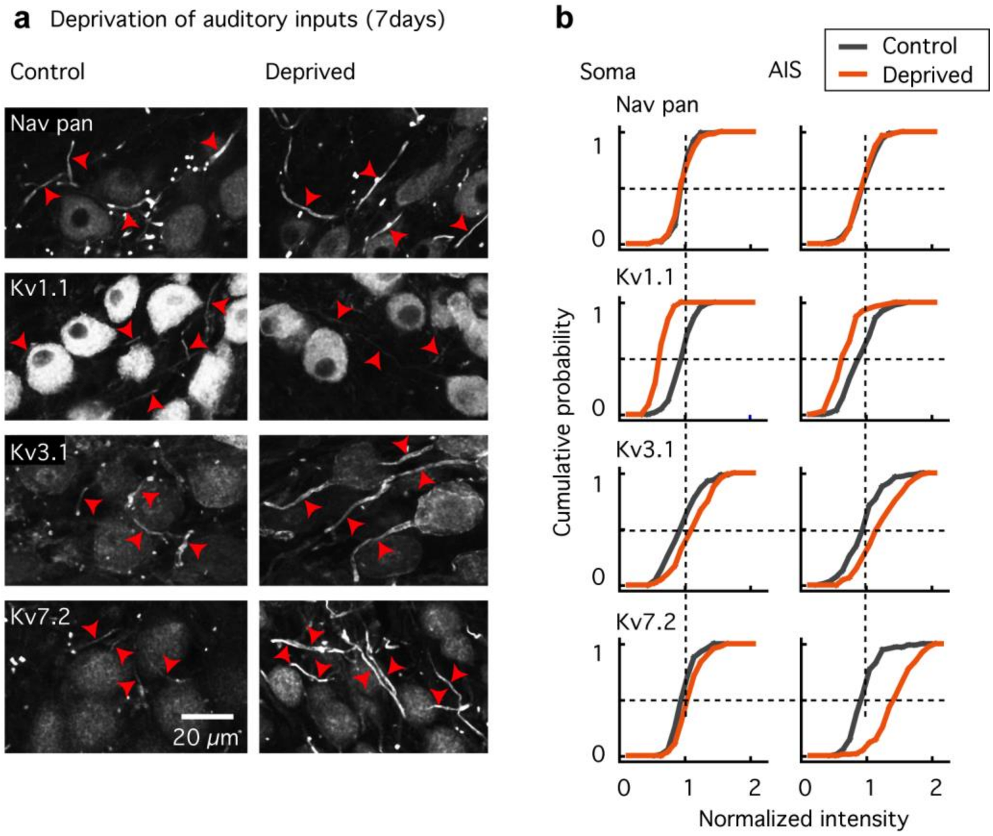

Supplementary Figure 1 Effects of auditory deprivation on Nav and Kv expressions at NM. (**a**) Immunosignals at control (left) and deprived (right) sides of NM within a slice at 7 days after auditory deprivation. Nav pan (top), Kv1.1 (upper middle), Kv3.1 (lower middle), and Kv7.2 (bottom). (**b**) Cumulative probability of signal intensity at the soma (left) and the AIS (right) in (**a**). Quantification was made in more than 100 cells from 5 animals (Methods).

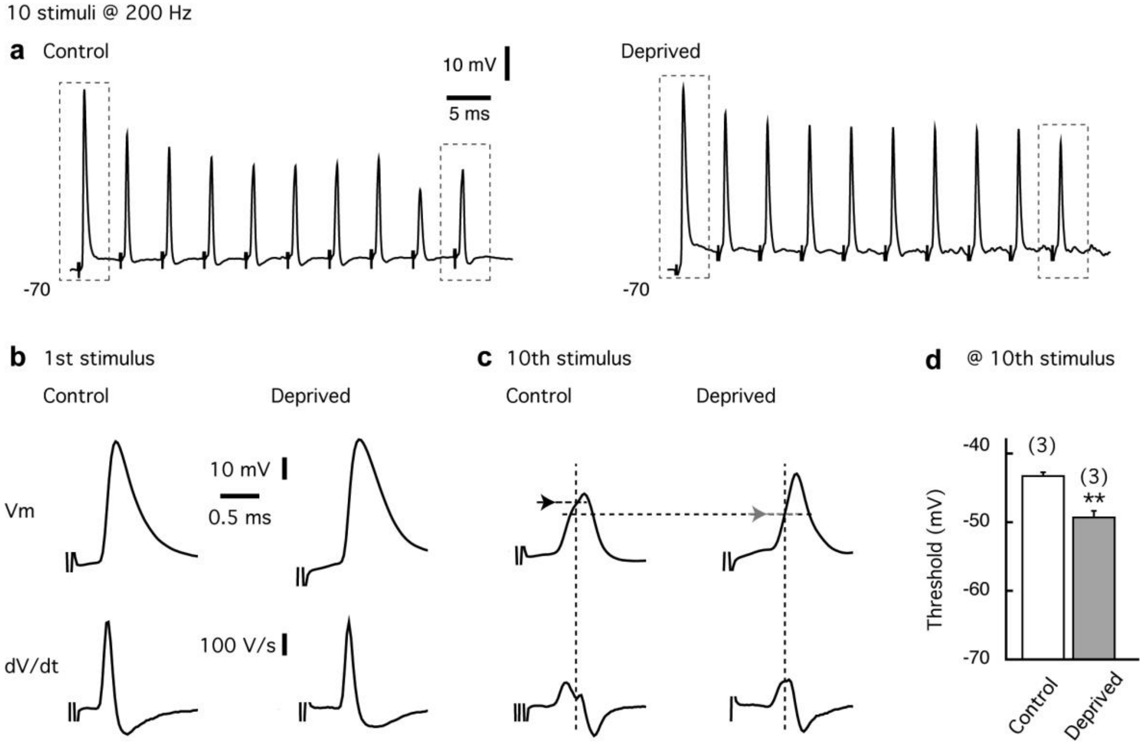

Supplementary Figure 2 APs in response to synaptic inputs. (**a**) APs were induced by electrically stimulating the auditory nerve at 200 Hz with a bipolar tungsten electrode. Control (left) and deprived (right). (**b**, **c**) Membrane potential (top) and its time derivative (bottom) at 1st (**b**) and 10th (**c**) stimuli in (**a**). Arrows indicate AP threshold. (**d**) AP threshold at 10th stimulus.
